# Hæmatology.—V

**Published:** 1910-05-28

**Authors:** 


### PATHOLOGY.

### HÆMATOLOGY.—V.

In the last article the value of the results of the Widal's reaction were discussed, and it was indicated that the bare laboratory result of positive or negative might vary considerably as to its clinical significance. The reaction in a typhoid fever case as a rule begins about the seventh day of the disease; it may, however, come as early as the fifth, or, on the other hand, may not appear until the third week or later, or even in some severe cases not at all. Muir and Ritchie, in their manual of bacteriology, state that in nearly 95 per cent. of the cases (of typhoid) the test can be satisfactorily obtained. They give as causes of possible error the following:—

(1) A bad race or strain and a bad state of the culture; (2) the serum of the person may naturally clump the bacilli; (3) a previous attack of typhoid with persistence of agglutination; (4) the case may be one caused by an allied bacillus (paratyphoid); (5) the disease may be due to something else and yet the serum clump the bacilli; and (6) the disease may be typhoid and yet no reaction occur.

There does not seem to be any definite agreement as to how long the reaction persists after an attack of typhoid fever, but in some instances it would seem to go on for years; this (the question of a previous attack) should therefore always be taken into account before coming to a diagnosis. Also of difficulty is Class 6, and such cases do occur. One such example, though clinically resembling typhoid, never gave the Widal reaction during life, though many different cultures were tried. After death an autopsy revealed typical typhoid lesions in the intestine, and an organism which answered all the tests of the typhoid bacillus was grown from the spleen.

*The Sedimentation Test.*—Small upright glass tubes are used for this test, the technique being very simple. Equal parts of the diluted serum and culture are placed in one of these (a control of culture alone should always be used as well) and left standing for 24 hours. If the reaction takes place at the end of that time there will be a deposit at the bottom of the tube, the upper part remaining clear, whereas if no reaction takes place the fluid will remain turbid throughout.

*To do the Reaction for Malta Fever.*—The agglutination reaction for Malta fever is carried out in much the same way as for typhoid, some minor points of difference having to be attended to however. The germ, the micrococcus melitensis, takes longer to grow than the typhoid bacillus, and one and a half to two days are required, as a rule, before the culture is ready. The emulsion is prepared in the same way, the young two days' old culture being employed. Different dilutions of the serum are necessary, the most useful ones being as 1-25, 1-50, 1-100, and 1-200. One of these higher dilutions should always be tried, as positive results with low dilutions alone, such as 1-25, have before now given rise to erroneous results. If properly carried out, the value of the test is undoubted.

### 7. The Bacteriological Examination of the Blood.

For the proper bacteriological examination of the blood a laboratory provided with the usual requisites is essential. The blood *per se* seems to be detrimental to the growth of germs in it, so in cultivating it has to be diluted as much as possible with the medium used. The procedure is as follows: The blood should always be drawn from a vein, the skin over the site of puncture having been previously carefully sterilised; it is then ejected from the syringe into a large bulk of broth, and this is incubated in the usual manner. If growth takes place, sub-cultures are made and the organism isolated. A recent method adopted for isolating the typhoid bacillus from the blood (it is said to be present there on the second or third day of the disease) is to pass some of the suspected blood into sterilised ox bile. The typhoid bacillus has a strong predilection for this fluid and grows in it quickly and readily. From here it can be sub-cultured later and put through the necessary tests to determine it.

(*To be continued.*)

